# New Biomarkers to Predict the Evolution of *In Situ* Breast Cancers

**DOI:** 10.1155/2014/159765

**Published:** 2014-08-26

**Authors:** S. Bravaccini, M. M. Tumedei, E. Scarpi, W. Zoli, C. Rengucci, L. Serra, A. Curcio, F. Buggi, S. Folli, A. Rocca, R. Maltoni, M. Puccetti, D. Amadori, R. Silvestrini

**Affiliations:** ^1^Biosciences Laboratory, Istituto Scientifico Romagnolo per lo Studio e la Cura dei Tumori (IRST), IRCCS, 47014 Meldola, Italy; ^2^Unit of Biostatistics and Clinical Trials, Istituto Scientifico Romagnolo per lo Studio e la Cura dei Tumori (IRST), IRCCS, 47014 Meldola, Italy; ^3^Pathology Unit, Morgagni-Pierantoni Hospital, 47121 Forlì, Italy; ^4^Breast Surgery Unit, Morgagni-Pierantoni Hospital, 47121 Forlì, Italy; ^5^Department of Medical Oncology, Istituto Scientifico Romagnolo per lo Studio e la Cura dei Tumori (IRST), IRCCS, 47014 Meldola, Italy; ^6^Pathology Unit, Santa Maria delle Croci Hospital, 48010 Ravenna, Italy

## Abstract

*Background.* Genomic studies have shown that gene expression profiles are similar in *in situ* (CIS) and invasive breast cancers, suggesting that several biofunctional modifications of the transformation process occur before or during the development of CIS lesion. *Methods.* We investigated 3 biomarkers in 44 patients with CIS: TG2 (transglutaminase 2), HJURP (Holliday junction recognition protein), and HIF-1α (hypoxia inducible factor-1 alpha). *Results.* TG2 was more highly expressed than the other two markers and significantly more so in stromal than in tumor cells. HIF-1α evaluation showed a higher expression in both tumor and stromal cells in patients with relapsed G3 tumors, indicating a potential role of this marker in CIS evolution. A greater than sevenfold higher risk of relapse (*P* = 0.050) was observed in patients highly expressing HJURP in stroma and a tenfold higher recurrence risk (*P* = 0.026) was seen in those with a higher stromal HIF-1α expression. An important increase in risk accuracy (AUC 0.80) was obtained when HIF-1α and HJURP were evaluated together. *Conclusions.* Despite the limited number of relapsed patients, we formulated some hypotheses on the factors responsible for malignant evolution and recurrence which are now being tested in a large case series with a longer follow-up.

## 1. Introduction 

The increasing use of mammographic screening has led to the diagnosis of a higher number of* in situ* or small invasive tumors. It has thus become important to acquire more information on biomarkers present in primary lesions or in their margins that are capable of predicting disease evolution in order to plan the most effective and least expensive personalized therapy.

In addition to the conventional steroid hormone receptors and cell proliferation markers, the role of several other biomolecular markers involved in the transition from* in situ* to invasive cancer has been investigated, for example, p27, microvessel density (MVD), and c-kit expression [1]. Genomic studies have recently demonstrated that gene expression profiles are similar in* in situ* and in invasive cancers [2–4], suggesting that numerous biofunctional modifications of the breast cancer transformation process arise before or during the development of an* in situ* lesion.

In the present study we aimed to search for new markers indicative of tumor aggressiveness and potentially capable of identifying patients with a high probability of relapse in order to maximize the benefit of tailored therapy. In particular, three molecular markers were investigated: TG2 (transglutaminase 2), HJURP (Holliday junction recognition protein), and HIF-1α (hypoxia inducible factor-1 alpha). TG2, an enzyme that catalyses protein cross-linking reactions, is a transcriptional target of HIF-1α that enhances the survival of hypoxic cells. HIF-1α protein is rapidly degraded under normoxic conditions by the ubiquitin-proteasome system controlled by an oxygen dependent degradation domain within HIF-1α. It has been seen that the accumulation of HIF-1α under hypoxia leads to stabilization of the protein [5].

It has been hypothesized that TG2 is involved in different biological processes, including apoptosis, membrane signaling, cell adhesion, and extracellular matrix formation. TG2 expression is also implicated in pathologic consequences, especially in certain cancer histotypes [6], and an increased expression of this marker has been observed in drug-resistant and metastatic breast cancer cells. At present, little is known about the underlying mechanism by which TG2 exerts antiapoptotic effect in cancer cells [7].

HJURP functions at the centromere level and is required for centromere protein A (CENP-A) centromeric localization, for the loading of new CENP nucleosomes, and for accurate chromosomal segregation. Hu et al. reported a direct relation between HJURP expression, poor clinical outcome, and higher sensitivity to radiotherapy in breast cancer patients [8]. HIF-1α is composed of transcriptional activator HIF-1*β* subunits constitutively expressed in the nucleus. It binds specific DNA sequences that regulate the transcription of genes involved in anaerobic metabolism, growth and survival of normal and transformed cells, and angiogenesis. Some authors have shown [9] that preoperative radiotherapy downregulates the nuclear expression of HIF-1α in rectal cancer. However, the impact of radiotherapy on TG2 and HIF-1α expression needs to be better defined by performing the same analyses on breast cancer patients treated with surgery only.

These markers have been investigated in cell lines [6] and to a lesser extent in* in situ* breast cancers, with the focus mainly on tumor cells and only occasionally on the stromal component. However, tumor microenvironment complexity has also been shown to play a role in breast cancer progression. Up to now, most of the results published have been obtained on cell lines [6] and only occasionally on* in situ* breast cancers.

The aim of the present study was to investigate the clinical significance of conventional and new markers (HJURP, HIF-1α, and TG2) in* in situ* breast cancer and in the tumor microenvironment and to assess any correlation with clinical outcome in patients homogeneously treated with surgery and postsurgery radiation therapy.

## 2. Materials and Methods

### 2.1. Ethics Statement

The study protocol was reviewed and approved by the Medical Scientific Committee of IRST IRCCS and all patients gave written informed consent to take part in the study.

### 2.2. Case Series

This retrospective study was performed on 70 patients selected from a case series consecutively enrolled from 2000 to 2008 at the Breast Unit of Morgagni-Pierantoni Hospital in Forlì in collaboration with the Cancer Prevention Unit and the Breast Surgery Unit of the same Hospital. Patients aged ≥18 years with a histological diagnosis of* in situ* breast cancer homogenously treated with quadrantectomy and radiotherapy were eligible. Biological determinations were limited to 44 patients due to a lack of representative material in the remaining cases. Twelve patients with recurrent breast cancer (cases) were matched to 32 nonrecurrent patients (controls) for age (≤56 and >56 years), grade (1-2, 3), and estrogen receptor (ER) (<10% and ≥10%) classes with an allocation ratio of 1 : 3. Recurrent disease was defined as a lesion occurring with any morphological type (*in situ* or invasive cancer) in the ipsilateral breast more than 12 months after the initial surgery. All the patients were followed up for at least 5 years. The original hematoxylin and eosin stained sections were reviewed by two pathologists who were asked to select pathologic inclusions representative of tumor tissue as well as their normal breast tissue counterparts. The Holland grading system, based on cytonuclear and architectural (cell polarization) differentiation, was used for ductal carcinoma* in situ* (DCIS). DCIS were classified into three types: type 1 (well), type 2 (intermediately), and type 3 (poorly) differentiated lesions. The study protocol was reviewed and approved by the local Ethics Committee and patients gave written informed consent to take part in the study.

### 2.3. Biomarker Determination

Biomarkers were determined by immunohistochemistry in tumor cells and in the surrounding stroma. Four-micron sections from neutral buffered formalin-fixed and paraffin-embedded tissue were mounted on positive-charged slides (Bio Optica, Milan, Italy), deparaffinized with xylene, and rehydrated in decreasing concentrations of ethanol to distilled water. Endogenous peroxidase activity in rehydrated deparaffinized sections was blocked by 3% hydrogen peroxide solution. For TG2, HJURP, and HIF-1α determinations, tissue sections were treated with epitope retrieval solution (0.01 M citrate buffer pH 6.0) in a water bath at 98.5°C for 40 min, followed by a 20-min cooling period at room temperature. The sections were treated with 3% bovine serum albumin in PBS for 20 min, after which they were incubated at room temperature for 30 min with TG2 (Epitomics, Burlingame, CA, USA), HJURP (Abcam Ltd., Cambridge, UK) and HIF-1α (Epitomics) antibodies diluted 1 : 500, 1 : 100, and 1 : 400 in antibody diluent with background reducing components (Dako Corporation, Carpinteria, CA, USA), respectively. Sections were counterstained with hematoxylin and washed several times with cold tap water. Biomarker positivity was semiquantitatively evaluated as the percentage ratio between immunopositive and total number of cells. All samples were evaluated by two independent observers. A disagreement of more than 10% of positive cells, observed in about 20% of cases, was resolved by consensus after joint review using a multihead microscope.

### 2.4. Statistical Analysis

Nonparametric ranking statistics (median test) were used to analyze the relationship between median values of TG2, HJURP, and HIF-1α and patient characteristics. Spearman's correlation was used to investigate the correlation between the different biomarkers considered as continuous variables. In the absence of internationally accepted cut-off values for TG2, HJURP, and HIF-1α, the cut-offs maximally discriminating between relapsed and nonrelapsed patients were identified using receiver operating characteristic (ROC) curve analysis. In the ROC curves, the true positive rates (sensitivity) were plotted against the false positive rates (1-specificity) for all classification points.

Logistic regression was used to estimate the odds ratio (OR) and 95% confidence intervals (95% CI) in order to evaluate the independent prognostic relevance of single biomarker expression (continuous or dichotomized variable) and patient status (relapsed/nonrelapsed). The accuracy of single or combined biomarkers, considered as continuous variables, was measured using the area under ROC curve (AUC), and the chi-squared test was performed to evaluate the differences in diagnostic accuracy. All *P* values were based on two-sided testing, and statistical analysis was carried out using SAS software, version 9.3 (SAS Institute, Cary, NC, USA).

## 3. Results

**Figure 1:**
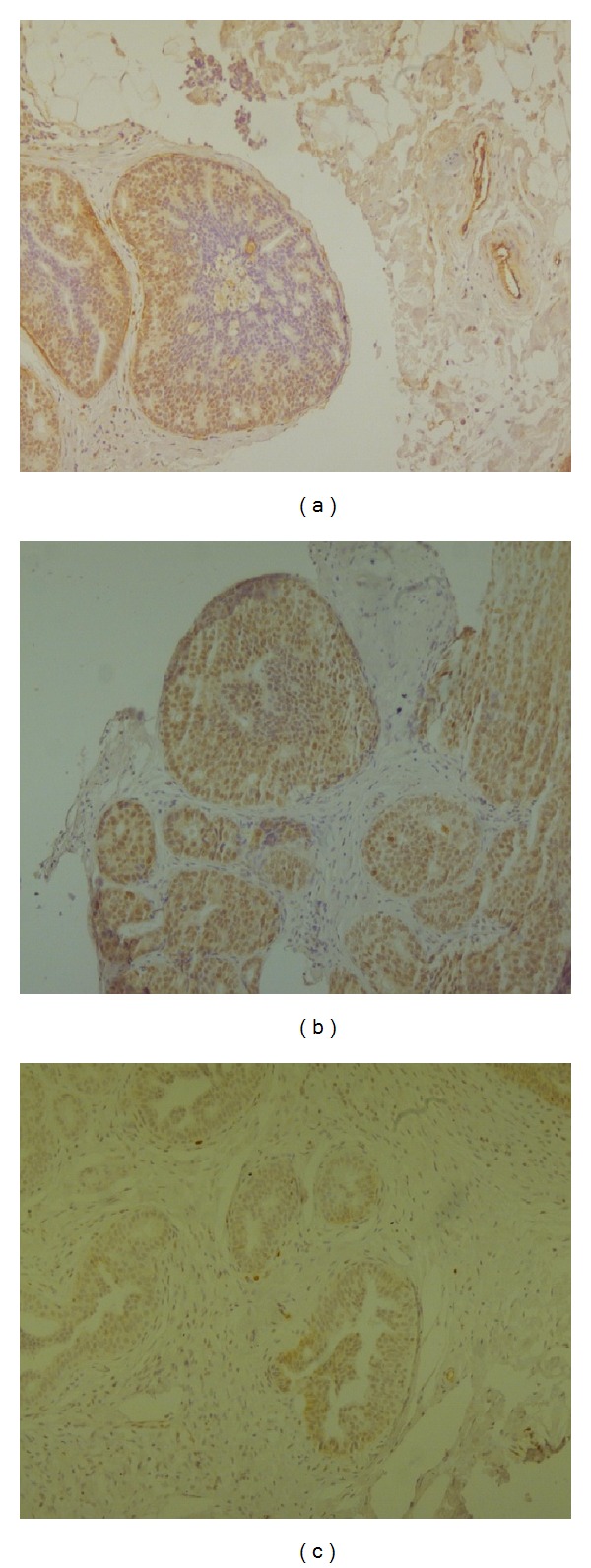
Immunohistochemical positivity of (a) TG2, (b) HJURP, and (c) HIF-1α in carcinoma* in situ* of the breast.

The median age of patients was 55 years (range 38–74 years); 77% had ER-positive and 59% progesterone receptor- (PgR-) positive lesions; 3 (7%) showed Holland type 1, 20 (45%) type 2, and 21 (48%) type 3 tumors. Expression levels of TG2, HJURP, and HIF-1α in tumor cells and in stroma are shown in Table 1 and Figure 1. TG2 was more highly expressed than the other two markers, significantlymore so in stromal than in tumor cells and with an evident, albeit nonsignificant, decrease in expression passing from grades (G)1-2 to G3 lesions. A lower expression of HJURP was observed in both tumor and stromal cells and was more evident in the latter, with no significant variation between G1-2 and G3 lesions. The lowest expression was observed for HIF-1α in both tumor and stromal cells, with a significantly higher expression in tumor cells in G3 with respect to G1-2 lesions. Interestingly, the expression of all three markers in the tumor cells and stroma of G3 lesions was statistically different.

The expression of the different markers was analyzed in relation to recurrence in patients with different grade lesions. No prognostic relevance of the different markers was observed in the overall series (Table 2). A breakdown analysis as a function of lesion grade showed a significantly higher expression of HJURP in stroma (*P* = 0.031) and an expression of borderline significance (*P* = 0.063) in tumor cells in patients with relapsed G1-2 lesions compared to disease-free cases. In patients with G3 lesions, relapse was significantly related to a higher HIF-1α expression in both tumor (*P* = 0.024) and stromal cells (*P* = 0.019).

Logistic regression analysis of single biomarker expression as a function disease recurrence showed no prognostic relevance of the markers when they were considered as continuous variables and a twofold (OR = 1.98), albeit nonsignificant (*P* = 0.067), risk of relapse when HIF-1α was expressed in the stromal component. The same analysis conducted on tumor cells showed higher TG2 values in nonrelapsed patients compared to relapsed cases (data not shown).

Conversely, considering stromal expression as a dichotomized variable and using cut-off values identified by ROC curve analysis, a more than sevenfold greater risk (*P* = 0.050) of relapse was observed for patients highly expressing HJURP, while a 10-fold higher risk (*P* = 0.026) of recurrence was seen for those highly expressing HIF-1α in stroma (Table 3). Conversely, these results were not significantly different when marker expression was evaluated in tumor cells rather than stroma.

In view of the lack of correlation observed among the different marker expression levels in the same tumor, we carried out combined marker analysis in an attempt to increase prognostic accuracy (Table 4). An AUC ranging from 0.56 to 0.63 was observed for single marker expression in both tumor and stroma compartments. No significant improvement in accuracy was obtained by combining TG2 with HJURP or HIF-1α in either tumor cells or in stroma, whereas an important increase in accuracy (AUC 0.80, 95% CI 0.63–0.97) was obtained when HIF-1 α and HJURP stromal expression was considered together. Finally, a slightly higher (0.84, 95% CI 0.70–0.98), albeit not significant, AUC was observed when the stromal expressions of all the three markers were combined.

## 4. Discussion 

One out of every five new breast cancers diagnosed each year is a ductal carcinoma* in situ* which represents an uncontrolled cell growth within the breast ducts. This histological type is known to be related to the risk of recurrence* in situ *lesions, but not to invasive recurrence, which is clinically more relevant [10]. The increasing use of breast-conserving surgery makes it essential to identify lesions at high risk of relapse or progression in order to offer patients the most suitable tailored therapy. The identification of biological markers that are predictive of relapse could thus help to optimize patient management.

A number of studies have attempted to define the events responsible for the transition from* in situ* to invasive lesions and to understand whether biofunctional changes characteristics of invasive breast cancer are already present in* in situ *breast disease [1, 2, 11–13]. Biofunctional changes include the loss of normal regulatory mechanisms that permit tumor cells to escape from the ducts and lobules in which they are physically contained [1]. However, many questions remain to be answered regarding the capacity of cells to acquire the invasive phenotype. Furthermore, as the standard therapeutic strategy for carcinoma* in situ* (CIS) is surgery and radiotherapy, more research is needed to identify the biomarkers present in primary lesions or lesion margins that can predict relapse or progression. Unfortunately, the few studies addressing this issue have produced conflicting results.

Although TG2, HJURP, and HIF-1α have proven to be important predictors of radiosensitivity in a number of tumor types, their role in the evolution of CIS has yet to be investigated [11]. The fact that these markers are highly expressed in infiltrating breast carcinoma [14, 15] prompted us to investigate their expression in CIS to better understand their functional role in the progression to the infiltrating phenotype. We also considered stromal cells surrounding the tumor following recent evidence of the role of the microenvironment in cancer progression.

The hypoxic microenvironment of tumor cells facilitates radio- and chemoresistance and the adaptation to hypoxia is regulated by HIF-1α. Our data showed a low expression of HIF-1α in tumor cells and an almost total absence in the surrounding stroma. However, a significantly higher expression was observed in the tumor cells of dedifferentiated tumors with respect to well and moderately differentiated lesions, suggesting a potential role of this marker in CIS evolution.

Alterations in the extracellular matrix affect host-tumor interactions, tumor growth, and metastasis formation. TG2 renders the extracellular matrix highly stable and resistant to proteolytic degradation. It is known that TG2 accumulates in the nucleus as a result of hypoxic and ischemic stress in neurons and that it interacts with hypoxia inducible factor-1*β* and suppresses HIF-dependent transcription [16]. TG2 expression in tumor samples has been associated with poor disease outcome, high drug resistance, and increased incidence of metastasis [14]. In agreement with other authors [16], we observed higher TG2 expression in stroma than in tumor cells in our overall case series and also in the different tumor grade subgroups. It has been suggested that increased expression of TG2 in stroma represents the host's attempt to restrict tumor growth and prevent it from spreading to distant sites [17].

HJURP mRNA level is a prognostic indicator of disease-free and overall survival in patients with infiltrating breast cancer and a predictive biomarker of radiosensitivity [8]. Furthermore, HJURP mRNA expression in invasive ductal carcinoma (IDC) is significantly higher than that present in normal breast ducts [8]. We observed that HJURP is more highly expressed in cancer cells than in stroma cells. Some authors have suggested that HJURP levels in normal breast tissue are capable of identifying patients who will show greater radiosensitivity, thus permitting measures to be taken to reduce potential side-effects [11]. In our case series, the prognostic relevance of single markers was only observed for tumor and stromal expression of HIF-1α in the G3 subgroupand for stromal HJURP expression in G1-2 lesions.

Our results revealed a different expression of the three markers in the two histologic components, that is, higher TG2 expression in stroma than in tumor cells and, conversely, higher HJURP and HIF-1α in tumor than in stromal cells, with a significant difference for all three marker expression levels in G3 lesions. Interestingly, the odds ratio analysis of these markers highlighted two immunohistochemical cut-off values for stromal HJURP and HIF-1α that identified patients with a sevenfold and tenfold higher risk of relapse, respectively. Conversely, no evidence of prognostic relevance of marker expression was observed in tumor cells. The combined analysis of HIF-1α and HJURP produced significantly higher predictive accuracy, whereas the combination of all three markers did not significantly increase accuracy in terms of AUC values.

Our next aim is to investigate whether the unfavorable prognostic impact of these markers reflects intrinsically more aggressive disease or tumor radioresistance. In the case of the latter, radiotherapy could be substituted with less intense hormone or chemotherapeutic treatment regimens.

## Figures and Tables

**Table 1:** Expression of different markers in tumor cells and stroma in relation to lesion grade (G).

Markers	Overall series *n* = 44 (median, range)	G1 + G2 *n* = 23 (median, range)	G3 *n* = 21 (median, range)	P*
TG2				
Tumor cells	10 (0–100)	20 (0–100)	3 (0–80)	0.195
Stroma	100 (0–100)	100 (20–100)	100 (0–100)	0.874
	*P* < 0.0001∗∗	*P* < 0.0001∗∗	*P *< 0.0001∗∗	
HJURP				
Tumor cells	3.5 (0–100)	3 (0–100)	4 (0–80)	0.582
Stroma	0 (0–80)	0 (0–80)	0 (0–70)	0.765
	*P* = 0.006∗∗	*P* = 0.065∗∗	*P* = 0.027∗∗	
HIF-1α				
Tumor cells	0 (0–70)	0 (0–10)	3 (0–70)	0.001
Stroma	0 (0–10)	0 (0–10)	0 (0–10)	0.732
	*P* = 0.231∗∗	*P* = 0.172∗∗	*P* = 0.013∗∗	

*P values refer to the difference between tumor cell and stroma marker expression in G1 and G2 with respect to G3 lesions.

**P values refer to the difference between tumor cell and stroma marker expression within the same tumor grade and in the overall series.

**Table 2:** Expression of different markers in tumor cells and stroma in relapsed and nonrelapsed patients.

Markers	Overall series			G1 + G2			G3		
	Nonrelapsed (median, range)	Relapsed (median, range)	*P *	Nonrelapsed (*n* = 17) (median, range)	Relapsed (*n* = 6) (median, range)	*P *	Nonrelapsed (*n* = 15) (median, range)	Relapsed (*n* = 6) (median, range)	*P *
TG2									
Tumor cells	15 (0–100)	3 (0–80)	0.306	20 (0–100)	11.5 (0–70)	0.532	10 (0–70)	2.5 (0–80)	0.463
Stroma	100 (0–100)	100 (70–100)	0.413	100 (20–100)	100 (70–100)	0.929	100 (0–100)	100 (100-100)	0.206
HJURP									
Tumor cells	3 (0–90)	8 (0–100)	0.555	2 (0–90)	80 (0–100)	0.063	10 (0–80)	0 (0–70)	0.192
Stroma	0 (0–70)	0 (0–80)	0.347	0 (0–70)	55 (0–80)	0.031	0 (0–70)	0 (0–3)	0.225
HIF-1α									
Tumor cells	0 (0–10)	1 (0–70)	0.137	0 (0–10)	0 (0-0)	0.439	0 (0–10)	7.5 (2–70)	0.024
Stroma	0 (0–2)	0 (0–10)	0.096	0 (0–2)	0 (0–10)	0.753	0 (0–2)	2 (0–10)	0.019

**Table 3:** Univariate analysis of different markers in tumor and stromal cells.

Markers	Tumor cells		Stroma	
	OR (95% CI)	*P *	OR (95% CI)	*P *
TG2	0.47 (0.08–2.69)	0.397	0.95 (0.24–3.76)	0.236
HJURP	4.59 (0.90–23.25)	0.066	7.11 (1.00–50.74)	0.050
HIF-1α	3.05 (0.58–15.97)	0.187	9.90 (1.32–74.42)	0.026

Cut-offs: TG2 tumor cells (<70 versus ≥70), stroma (≥80 versus <80); HJURP tumor cells (≥70 versus <70), stroma (≥30 versus <30); HIF-1α tumor cells (≥4 versus <4), stroma (≥2 versus <2).

**Table 4:** Area under the curve (AUC) of single and combined markers in tumor and stromal cells.

Markers	Tumor cells AUC (95% CI)	*P*	Stroma AUC (95% CI)	*P*
TG2	0.60 (0.40–0.81)	—	0.56 (0.44–0.68)	—
HJURP	0.56 (0.34–0.78)	—	0.58 (0.39–0.77)	—
HIF-1α	0.63 (0.45–0.81)	—	0.63 (0.45–0.81)	—
TG2 + HJURP	0.62 (0.41–0.82)	0.202	0.65 (0.49–0.82)	0.060
TG2 + HIF-1α	0.65 (0.45–0.85)	0.682	0.68 (0.49–0.86)	0.396
HIF-1α + HJURP	0.72 (0.55–0.90)	0.317	0.80 (0.63–0.97)	0.071
HIF-1α + TG2 + HJURP	0.73 (0.57–0.90)	0.689	0.84 (0.70–0.98)	0.175
